# Association between glucose-to-lymphocyte ratio and in-hospital mortality in intensive care patients with sepsis: A retrospective observational study based on Medical Information Mart for Intensive Care IV

**DOI:** 10.3389/fmed.2022.922280

**Published:** 2022-08-24

**Authors:** Shaoyan Cai, Qinjia Wang, Chuzhou Ma, Junheng Chen, Yang Wei, Lei Zhang, Zengqiang Fang, Liangjie Zheng, Chunming Guo

**Affiliations:** ^1^Department of Anesthesiology, Shantou Central Hospital, Shantou, China; ^2^Department of Gastroenterology, The First Affiliated Hospital of Shantou University, Shantou, China

**Keywords:** glucose-to-lymphocyte ratio, sepsis, MIMIC-IV, in-hospital mortality, non-linearity, intensive care unit

## Abstract

**Background:**

This study aimed to evaluate the association between the glucose-to-lymphocyte ratio (GLR) and in-hospital mortality in intensive care unit (ICUs) patients with sepsis.

**Methods:**

This is a retrospective cohort study. Patients with sepsis from the Medical Information Mart for Intensive Care-IV (MIMIC-IV) database had their baseline data and in-hospital prognosis retrieved. Multivariable Cox regression analyses were applied to calculate adjusted hazard ratios (HR) with 95% confidence intervals (CI). Survival curves were plotted, and subgroup analyses were stratified by relevant covariates. To address the non-linearity relationship, curve fitting and a threshold effect analysis were performed.

**Results:**

Of the 23,901 patients, 10,118 patients with sepsis were included. The overall in-hospital mortality rate was 17.1% (1,726/10,118). Adjusted for confounding factors in the multivariable Cox regression analysis models, when GLR was used as a categorical variable, patients in the highest GLR quartile had increased in-hospital mortality compared to patients in the lowest GLR quartile (HR = 1.26, 95% CI: 1.15–1.38). When GLR was used as a continuous variable, each unit increase in GLR was associated with a 2% increase in the prevalence of in-hospital mortality (adjusted HR = 1.02, 95% CI: 1.01–1.03, *p* = 0.001). Stratified analyses indicated that the correlation between the GLR and in-hospital mortality was stable. The non-linear relationship between GLR and in-hospital mortality was explored in a dose-dependent manner. In-hospital mortality increased by 67% (aHR = 1.67, 95% CI: 1.45–1.92) for every unit GLR increase. When GLR was beyond 1.68, in-hospital mortality did not significantly change (aHR: 1.04, 95% CI: 0.92–1.18).

**Conclusion:**

There is a non-linear relationship between GLR and in-hospital mortality in intensive care patients with sepsis. A higher GLR in ICU patients is associated with in-hospital mortality in the United States. However, further research is needed to confirm the findings.

## Background

Sepsis is a serious public health concern worldwide. Sepsis is a life-threatening organ dysfunction caused by dysregulated host systemic inflammation and immune response to infection (1, 2). Despite advances in the recognition and management of clinical sepsis (3), morbidity and mortality remain high (4, 5), with sepsis-related deaths accounting for 19.7% of global deaths (6). To date, the exact mechanism of sepsis remains unclear but is widely hypothesized.

Many clinical studies consider sepsis to be a host-mediated systemic inflammatory response to infection, and evidence of dysregulated immune cell activation and host response has been observed in patients with severe sepsis (7, 8). In addition, some systemic inflammatory biomarkers have been reported to be associated with sepsis and poor prognosis, including neutrophil-lymphocyte ratio (NLR) (9–11), platelet–lymphocyte ratio (PLR) (12), lymphocyte–monocyte ratio (LMR) (13), and red cell distribution width (RDW) (14–16). The loss and dysfunction of immune cells are considered the main factors for secondary infections and poor outcomes in patients with sepsis. Therefore, alterations in immune cell number and function may be related to mortality in patients with sepsis (17). Lymphocytes are one of the primary effector cells involved in the systemic inflammatory response of sepsis. Extensive lymphocyte apoptosis is a key contributor to the development of the immunosuppressive phase of sepsis (18). Their profound role in immunosurveillance, which may protect the host from sepsis development and impaired immune system, has been reported to be associated with poor prognosis in patients with sepsis (9). Consequently, lymphocyte count indicating the state of the immune system appears to predict the outcomes of patients with sepsis (18).

In addition, numerous studies have demonstrated an association between failure to control hyperglycemia and adverse outcomes in patients in the intensive care unit (ICU), including death, nosocomial infection, wound complications, prolonged ICU stay, and an increased incidence of critical illness neuropathy (19). Acute hyperglycemia is an independent risk factor for in-hospital mortality in critically ill patients with sepsis (20).

The imbalance between these two indicators is reflected in the changes in the glucose-to-lymphocyte ratio (GLR). In this case, increased GLR indicates an imbalance in glucose regulation and immune responses (21). This imbalance leads to organ failure, metabolic problems, immune deficiencies, and oxygen supply and demand mismatch, all leading to death (22). There is growing evidence that elevated glucose levels and decreased lymphocyte counts are strongly associated with sepsis severity (11, 23). GLR may reflect the synergistic effect of hyperglycemia and immune dysfunction in critically ill patients (24). In addition, an increased GLR has been associated with poor prognosis in a range of disease cases, such as gallbladder cancer (25), pancreatic cancer (26), acute pancreatitis (27), and acute kidney injury (24). However, previous studies have not evaluated the prognostic relationship between biomarkers combined with glucose and lymphocyte counts in patients with sepsis. This study sought to assess the relationship between the GLR and hospital outcomes in patients with sepsis, an index that includes both glucose levels and systemic inflammation and may provide a new basis and reference for the clinical management of sepsis.

## Materials and methods

### Data source

We enrolled patients with sepsis from the MIMIC-IV (Medical Information Mart for Intensive Care IV, version 1.0) (28) database of the Massachusetts Institute of Technology (MIT). More than 70,000 adult patients were admitted to the intensive care unit (ICU) of Beth Israel Deaconess Medical Center in Boston between 2008 and 2019. Informed consent was waived because the data were obtained from publicly available sources. One author, Shaoyan Cai, obtained full access to the database and completed the data extraction (certification number 46658933). Strengthening the Reporting of Observational Studies in Epidemiology guidelines (29) was used to conduct this study.

### Participants

Patients aged >18 years who fulfilled the Sepsis-3 criteria (1) were eligible for our study. Sepsis was defined as an increase of ≥2 points in the sequential organ failure assessment (SOFA) score, plus documented or suspected infection (1, 30).

Septic shock was defined as (ICD) code 78552 (9th revision) and ICD code R6521 (10th revision). The diagnosis of diabetes was based on ICD-9. If patients were admitted to the ICU more than once, we only adopted the date of their first ICU admission (31).

### Variates

Variables considered confounders of sepsis outcomes based on existing literature and clinical judgment were included (23, 32), except glucose and lymphocytes count because of their collinearity with GLR.

Demographic and admission information: age, sex, ethnicity, insurance, weight, Charlson comorbidity index (CCI), and severity at admission, as measured by the Acute Physiology Score (APS) III score and SOFA score.

Vital signs: Heart rate, mean arterial pressure (MAP), and SPO2 at ICU admission.

Interventions: Mechanical ventilation, renal replacement treatment (RRT), and vasopressor agent use during the first 24 h of ICU admission.

Laboratory results: Glucose, lymphocyte count, hemoglobin, white blood cell (WBC) count, platelet count, neutrophil count, lactate, and pH.

GLR was calculated using the serum blood glucose (mmol/L)/lymphocyte count (× 109/L).

If the above data were tested multiple times within 24 h, we chose the first set of parameters.

### Outcome

The outcome was in-hospital mortality, which is defined as survival status at hospital discharge. Patients without any outcome information were excluded from the final cohort.

### Statistical analysis

Descriptive analysis was performed for categorical variables to assess the significance of differences between groups stratified by GLR quartiles (<0.43; 0.43–0.78; 0.78–1.56; ≥1.56) using the Kruskal–Wallis test or one-way analysis of variance. Baseline characteristic data are presented as proportions (%) and were compared using chi-square tests for categorical variables. Normally distributed continuous data are presented as mean ± standard deviation (SD) and compared using Student’s *t*-test between groups, while skewed distribution data are presented as the median and interquartile range (IQR) and compared using the Wilcoxon rank-sum test.

A multivariate Cox proportional hazard model was used to assess the independent association between the GLR and in-hospital mortality. We constructed three models: Model 1, adjusted only for age and sex. Model 2 was additionally adjusted for ethnicity, weight, MAP, hazard ratio (HR), SPO2, hemoglobin, platelet (PLT), WBC, lactate, and pH. Model 3 was additionally adjusted for SOFA score, APS III score, ventilator use, diabetes, CCI, vasopressin usage, and neutrophil count. In all models, linear trends were tested using GLR quartiles as categorical variables by assigning the median values of the quartiles to the variable.

A Cox proportional hazards regression model was used to assess the non-linear relationship between GLR and the outcome of sepsis. Based on the curve fitting (restricted cubic spline), we conducted a two-piecewise linear regression model to identify threshold effects, if a non-linear correlation was observed. Threshold levels of GLR were determined using a recursive method, and a maximum likelihood model was yielded.

A sensitivity analysis was performed to ensure the robustness of the data analysis. GLR was transformed into a categorical variable and a *p*-value for the trend was calculated. The purpose of this test was to validate the results of treating the GLR as a continuous variable and to determine the possibility of non-linearity.

Hospital survival was assessed using Kaplan–Meier survival curves according to GLR quartiles and evaluated using the log-rank test.

Stratified and interaction analyses were applied based on sex (male or female), age (<65 or ≥65 years), diabetes (yes or no), ventilator use (yes or no), and RRT use (yes or no). Subgroup analyses were adjusted for relevant covariates (age, sex, ethnicity, weight, MAP, HR, SPO2, hemoglobin, PLT, WBC, lactate, pH, SOFA score, APS III, ventilator use, diabetes, CCI, and vasopressin use).

The percentages of covariates with missing data were less than 30% for all analyses. The missing values of the covariates were imputed *via* multiple imputations. We created and analyzed three datasets together. To assess the robustness of the findings, we applied sensitivity analysis of patients after excluding missing data from the study (Supplementary Table 1).

Data analyses were performed using packages R 4.1.2 (The R Foundation)^1^ software and Free Statistics software versions 1.5. *P*-values < 0.05 were considered significant.

## Results

### Population

**FIGURE 1:**
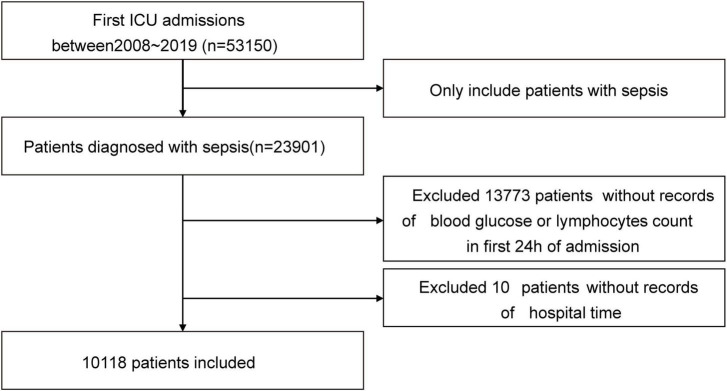
Flowchart of study patients.

In total, 23,901 patients were identified according to the sepsis-3 criterion. Of these, 13,343 patients without GLR values and in-hospital time were excluded, and 10,118 with sepsis were included in the final cohort (Figure 1 shows a flow chart).

### Baseline characteristics

The basic demographic characteristics of all selected patients are summarized in Table 1, stratified by GLR quartile. In general, the age of all participants was 65.8 ± 16.3 years old, and approximately 42.1% were female. The in-hospital mortality rate was 20.1% (480/2,383). Participants in the highest group of GLR (Q4) had higher values for age, heart rate, hemoglobin, platelet, WBC, neutrophil, lactate, glucose, APS III score, CCI, SOFA score, and were more likely to have diabetes, RRT, vasopressin use, and death than those in the other groups. The opposite patterns were observed for SPO2, pH, lymphocytes, and insurance for medical aid.

**TABLE 1:** Baseline characteristics of participants and outcome parameters.

Variables	All patients	Q1	Q2	Q3	Q4	*P*-value
					
		(GLR < 0.43)	(0.43 ≤ GLR < 0.78)	(0.78 ≤ GLR < 1.56)	(GLR ≥ 1.56)	
N	10118	2447	2576	2546	2549	
Age(year)	65.8 ± 16.3	64.7 ± 16.0	65.1 ± 16.3	66.1 ± 17.0	67.3 ± 15.8	< 0.001
Female, n (%)	4262 (42.1)	1004 (41)	991 (38.5)	1139 (44.7)	1128 (44.3)	< 0.001
Ethnicity, white, n (%)	6643 (65.7)	1630 (66.6)	1724 (66.9)	1640 (64.4)	1649 (64.7)	0.188
Insurance, Medicaid, n (%)	5602 (55.4)	1429 (58.4)	1476 (57.3)	1378 (54.1)	1319 (51.7)	< 0.001
weight(kg)	83.7 ± 23.7	83.0 ± 21.7	84.2 ± 23.4	83.9 ± 24.3	83.8 ± 25.2	0.295
**Vital Signs**						
Heart rate (bpm)	87.8 ± 16.3	85.1 ± 15.3	86.7 ± 15.3	89.1 ± 16.8	90.2 ± 17.3	< 0.001
MAP (mmHg)	75.8 ± 9.9	75.6 ± 9.2	75.8 ± 9.8	76.1 ± 10.1	75.9 ± 10.5	0.246
SPO2 (%)	96.8 ± 2.6	97.2 ± 2.4	96.9 ± 2.6	96.7 ± 2.3	96.4 ± 2.9	< 0.001
**Laboratory results**						
Hemoglobin (g/L)	10.4 ± 1.9	10.1 ± 1.7	10.4 ± 1.8	10.5 ± 2.0	10.6 ± 2.1	< 0.001
Platelet (× 1012)	171.0 (121.5, 239.0)	145.5 (109.5, 197.5)	167.0 (124.5, 231.1)	186.5 (128.5, 256.6)	195.0 (132.5, 272.5)	< 0.001
WBC(× 109/L)	12.5 (8.9, 16.9)	9.7 (6.6, 13.1)	11.9 (8.7, 15.3)	13.3 (9.7, 17.7)	15.7 (11.7, 21.5)	< 0.001
Neutrophil (× 109/L)	9.9 (6.6, 14.1)	6.7 (4.3, 9.4)	9.6 (6.8, 12.5)	10.8 (7.8, 14.7)	13.8 (9.8, 18.6)	< 0.001
Lactate (mmol/L)	2.6 ± 2.2	2.3 ± 1.8	2.4 ± 1.9	2.7 ± 2.4	3.1 ± 2.4	< 0.001
pH	7.4 ± 0.1	7.4 ± 0.1	7.4 ± 0.1	7.3 ± 0.1	7.3 ± 0.1	< 0.001
Glucose (mmol/L)	7.2 (6.1, 9.1)	6.2 (5.4, 7.1)	6.8 (5.9, 7.9)	7.7 (6.5, 9.6)	9.3 (7.3, 12.2)	< 0.001
Lymphocytes(× 109/L)	9.7 (5.3, 16.0)	21.4 (17.4, 27.7)	12.0 (10.0, 14.5)	7.3 (5.9, 9.2)	3.4 (2.1, 5.0)	< 0.001
GLR	0.8 (0.4, 1.6)	0.3 (0.2, 0.4)	0.6 (0.5, 0.7)	1.1 (0.9, 1.3)	2.6 (2.0, 3.9)	< 0.001
**Score system, points**						
CCI	5.8 ± 2.9	5.3 ± 2.8	5.5 ± 2.9	5.9 ± 3.0	6.3 ± 3.0	< 0.001
APS III score	58.0 ± 27.6	49.2 ± 25.5	53.3 ± 25.8	61.0 ± 27.2	68.3 ± 27.9	< 0.001
SOFA score	3.9 ± 2.2	3.8 ± 2.0	3.7 ± 2.0	3.9 ± 2.3	4.1 ± 2.4	< 0.001
**Interventions**						
Ventilator use, n (%)	5202 (51.4)	1259 (51.5)	1363 (52.9)	1284 (50.4)	1296 (50.8)	0.304
Diabetes, n (%)	3058 (30.2)	617 (25.2)	675 (26.2)	793 (31.1)	973 (38.2)	< 0.001
RRT, n (%)	565 (5.6)	85 (3.5)	95 (3.7)	166 (6.5)	219 (8.6)	< 0.002
Vasopressin use, n (%)	996 (9.8)	148 (6)	204 (7.9)	263 (10.3)	381 (14.9)	< 0.001
death, n (%)	1726 (17.1)	227 (9.3)	326 (12.7)	479 (18.8)	694 (27.2)	< 0.001

Data are presented as the mean ± standard deviation (SD), median (IQR) for skewed variables, and numbers (proportions) for categorical variables.

bpm, beats per minute; MAP, mean arterial pressure; WBC, white blood count; GLR, glucose-to-lymphocyte ratio; CCI, Charlson comorbidity index; APS III, Acute Physiology Score III; SOFA, Sequential Organ Failure Assessment; RRT, renal replacement treatment.

### Multivariable Cox regression analysis

In this study, we constructed three models to analyze the independent effects of the GLR on in-hospital mortality (multivariate Cox regression model; Table 2). The effect sizes (HRs) and 95% confidence intervals were listed. We observed that the HRs were robust between the unadjusted and adjusted models in all three models (*p* < 0.05). In the unadjusted model, the effect size of GLR for in-hospital mortality means that a difference of one unit of GLR is associated with an in-hospital mortality difference increased by 11% (HR = 1.11, 95% CI: 1.10–1.12). In the minimum-adjusted model (Model 1), with an increase in the GLR of one unit, the in-hospital mortality difference increased by 11% (HR = 1.11, 95% CI 1.1–1.12). In the fully adjusted model (Model 3) (adjusted covariates of age, sex, ethnicity, weight, MAP, HR, SPO2, hemoglobin, PLT, WBC, lactate, pH, SOFA score, APS III, ventilator use, diabetes, CCI, vasopressin usage, and neutrophil count) for each additional unit of GLR, in-hospital mortality difference increased by 2% (HR = 1.02, 95% CI 1.01–1.03). For further sensitivity analysis, the continuous variable GLR was converted into a categorical variable (quartile of GLR), of which the first category GLR (Q1) was used as a baseline reference. Patients in the highest GLR quartile had increased in-hospital mortality compared to patients in the lowest GLR quartile (HR = 1.26, 95% CI 1.15–1.38). The *P* for the trend in the fully adjusted model for GLR as a categorical variable was the result when GLR was a continuous variable. Moreover, the trend for effect size in the different GLR groups was equidistant.

**TABLE 2:** Multivariable Cox regression to assess the association of GLR with in-hospital mortality.

Variable	Unadjusted		Model 1		Model 2		Model 3	
				
	HR_95CI%	*P*-value	HR_95CI%	*P*-value	HR_95CI%	*P*-value	HR_95CI%	*P*-value
GLR	1.11 (1.1∼1.12)	< 0.001	1.11 (1.1∼1.12)	< 0.001	1.06 (1.05∼1.07)	< 0.001	1.02 (1.01∼1.03)	0.004
GLR4								
Q1(GLR < 0.43)	1(Ref)		1(Ref)		1(Ref)		1(Ref)	
Q2(0.43 ≤ GLR < 0.78)	1.23 (1.12∼1.36)	< 0.001	1.23 (1.12∼1.36)	< 0.001	1.18 (1.07∼1.3)	0.001	1.2 (1.08∼1.32)	0.001
Q3(0.78 ≤ GLR < 1.56)	1.6 (1.46∼1.76)	< 0.001	1.57 (1.43∼1.72)	< 0.001	1.34 (1.22∼1.47)	< 0.001	1.23 (1.12∼1.35)	< 0.001
Q4(GLR ≥ 1.56)	2.33 (2.14∼2.55)	< 0.001	2.25 (2.06∼2.46)	< 0.001	1.6(1.46∼1.75)	< 0.001	1.3(1.185∼1.43)	< 0.001
*P* for trend.test		< 0.001		<0.001		< 0.001		<0.001

GLR, glucose-to-lymphocyte ratio.

Model 1 = Adjust for (Age + sex).

Model 2 = Model 1 + (ethnicity + weight + MAP + HR + SPO2 + hemoglobin + PLT + WBC + lactate + pH).

Model 3 = Model 2 + (SOFA score + APS III + ventilator use + diabetes + CCI + vasopressin use + neutrophil).

### Kaplan–Meier curves

**FIGURE 2:**
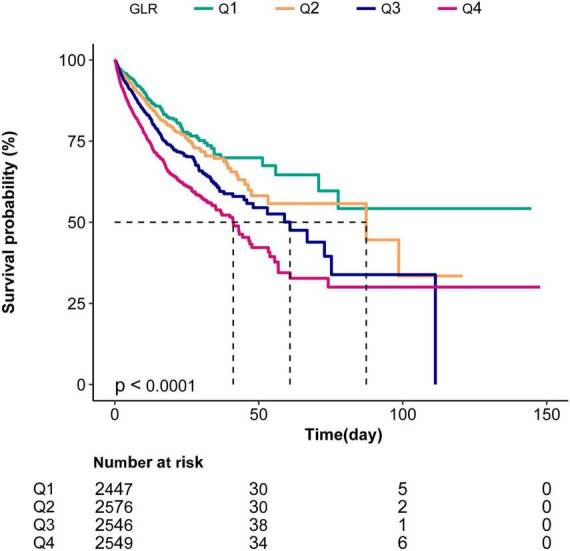
Kaplan–Meier curves indicating the association between the GLR and in-hospital mortality of sepsis patients. Q1, GLR < 0.43; Q2,0.43 ≤ GLR < 0-78; Q3,0.78 ≤ GLR < 1.56; Q4, GLR ≥ 1.56.

The Kaplan–Meier curve demonstrated that the in-hospital survival of the highest GLR quantile (Q4) patients was the lowest of all groups, which declined with declining baseline GLR (log-rank test: *p* < 0.0001; Figure 2).

### Subgroup analysis

**FIGURE 3:**
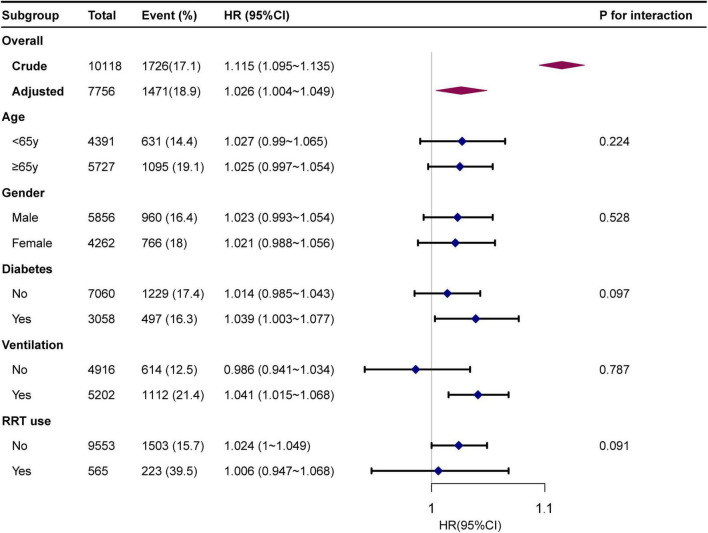
Forest plot for subgroup analysis for the association between GLR and in-hospital mortality. Each stratification adjusted for all the factors of model 3 in the Multivariable cox regression, except for the stratification factor itself.

Subgroup analyses indicated no significant interaction in the subgroup analysis (all *p*-values for interaction were >0.05; Figure 3).

### The analyses of the non-linear relationship

**FIGURE 4:**
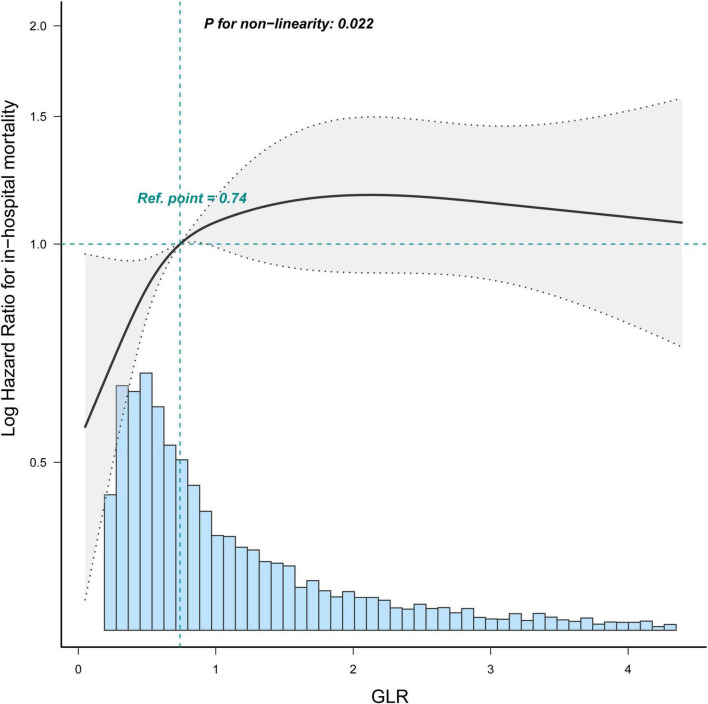
Restricted cubic spline shows the association between GLR and in-hospital mortality of sepsis patients. Data were fit by a Cos proportional hazard regression model based on restricted cubic splines. GLR was entered as continuous variable. Data were adjusted for all the factors of model 3 of Table 3. The curves line and shaded areas around depict the estimated values and their corresponding 95% confidence intervals. Only 95% of the data is displayed.

Restricted cubic spline (Figure 4) showed that the relationship between GLR and in-hospital mortality was non-linear after adjusting for related confounding factors. Because the *P* for the log-likelihood ratio test was <0.05, we chose the two-piecewise Cox proportional hazard model for fitting the association between GLR and in-hospital mortality. By the two-piecewise Cox proportional hazard model and recursive algorithm, we calculated the inflection point was 1.68. It was shown that stronger positive association between GLR and in-hospital mortality within the inflection point of 1.68. In-hospital mortality increased by 67% (aHR = 1.67, 95% CI: 1.45–1.92) for every unit GLR increase. When GLR was beyond 1.68, in-hospital mortality did not significantly change (aHR: 1.04, 95% CI: 0.92–1.18; Table 3).

**TABLE 3:** Threshold effect analysis of the relationship between GLR and in-hospital mortality of patients with sepsis.

Threshold of GLR	HR 95CI%	*P*-value
< 1.68	1.67 (1.45,1.92)	< 0.001
≥ 1.68	1.04 (0.92,1.18)	0.5223
Likelihood Ratio test	-	< 0.001

Data were adjusted for all the factors of Model 3 of Table 2.

## Discussion

This study evaluated the association of GLR, a combination of blood glucose levels and lymphocyte count, with in-hospital mortality after adjusting for the variables in a population-based analysis. Our findings indicate that an elevated GLR is associated with higher in-hospital mortality. Furthermore, as a continuous or categorical variable, GLR was positively associated with in-hospital mortality in intensive care patients with sepsis in the United States. Besides, the inflection point of GLR was 1.68, and we found the trend of HR on the two sides of the inflection point was not consistent. The result suggested a turning point effect on the independent association between GLR and in-hospital mortality.

Sepsis is characterized by systemic and organ-specific metabolic changes. Altered oxygen consumption, elevated circulating substrate levels, impaired glucose and lipid oxidation, and mitochondrial dysfunction are associated with organ dysfunction and adverse outcomes in animal models and patients (33). Sepsis can lead to a loss of glucose homeostasis, and the resulting hyperglycemia adversely affects immune function and metabolism, leading to poor outcomes (34, 35). The mechanisms that lead to glucose dysregulation are complex. Elevated blood glucose levels tend to reduce membrane fluidity, which impedes polymorphonuclear leukocyte (PMN) function, leading to reduced phagocytosis, intracellular killing, suboptimal migration, and chemotaxis (20, 36, 37). In addition, the neuroendocrine stress response can increase adrenal cortex secretion by 10 times, including excessive glycogenolysis, gluconeogenesis, and insulin resistance (38).

A low lymphocyte count may also be associated with a shortened survival time in sepsis (22). Clinical studies have shown that lymphocyte counts in the blood decrease during sepsis and remain low for up to 28 days (39, 40). Although the absolute lymphocyte counts of sepsis survivors and non-survivors were severely decreased at the onset of sepsis, lymphocyte counts recovered in survivors, while absolute lymphocyte counts remained persistently low in non-survivors (41). Various anti-inflammatory cytokines released into the bloodstream can induce immunosuppression and lead to massive lymphocyte apoptosis (42). Lymphopenia is a common marker of sepsis-induced immunosuppression, as it prevents microbial clearance and induces severe infections, which are the leading causes of sepsis-related death (39). Apoptosis-induced lymphocytopenia often occurs in sepsis and severe injuries, including major surgery, burns, and trauma. As active lymphocytes migrate to inflammatory areas, lymphocyte apoptosis increases (43). This process begins immediately after the potential damage occurs. The severity and duration of lymphocytopenia are associated with poor clinical outcomes. The severity and duration of lymphopenia are associated with poor clinical outcomes. Extensive apoptosis of lymphocytes occurs in lymphoid (lymph nodes, thymus, and spleen) and other organs (44) leading to impaired immune cell activity, which is a key contributor to the development of the immunosuppressive phase of sepsis and plays a direct or indirect role in injury-induced immune paralysis (45).

The exact mechanism underlying the association between elevated GLR levels and poor prognosis in patients with sepsis is unclear. Recently, several researchers have been interested in biomarkers that combine blood glucose levels and inflammatory indicator lymphocytes to predict the prognosis of certain diseases. Navarro suggested that the preoperative GLR was an independent predictor of overall survival (OS) and disease-free survival (DFS) after surgery for T2 gallbladder cancer. This is the first report of the predictive value of GLR (25). As an easily available biomarker, Chen et al. reported that GLR was an independent predictor of in-hospital mortality in critically ill patients with acute pancreatitis. They combined GLR with other clinical characteristics of acute pancreatitis to construct nomograms with favorable predictive performance for in-hospital mortality (27). Two other studies showed that GLR is an independent predictor of prognosis in patients with pancreatic cancer (26, 46). Preoperative GLR was also a promising predictor of acute kidney injury after cardiac surgery in ICU patients (24). Therefore, it is worth considering that GLR may reflect a synergistic effect of immunocompromise and hyperglycemia in sepsis.

To the best of our knowledge, this is the first report of an independent association between GLR and in-hospital mortality in ICU patients with sepsis. This study could help establish diagnostic or predictive models of in-hospital sepsis mortality in future research.

Our study has several strengths. First, the study used real-world data for a large and diverse population. Second, strict statistical adjustment was used to minimize susceptibility to potential residual confounders in this retrospective observational study. Third, we considered the target independent variables as both continuous and categorical variables. With this approach, contingency in the data analysis was reduced, and the robustness of the results was enhanced. Fourth, the non-linear processing of the study is a major improvement compared to former studies. Finally, the effect modifier factor analysis improved data usage and yielded more robust results in different subgroups.

There are some noteworthy limitations to this study. First, in the MIMIC-IV database, we could not obtain data on procalcitonin and organ functions, and other residual confounders potentially exist, as in all retrospective analyses. Some patients with sepsis were excluded from our study because of the lack of necessary data, which may have led to bias in the study results. Second, the influence of antibiotic use on results was not considered. We believe that this is an important subject and will be the objective of our future research. Third, our research subjects were intensive care patients with sepsis. Therefore, the universality and extrapolation of research are lacking. Moreover, GLR values changed dynamically during hospitalization. However, the GLR value used in the present study was not calculated based on the date of the onset of sepsis but on the first day of admission to the ICU or hospital. Therefore, this may have caused bias in the results. Finally, it was a retrospective study based on the MIMIC-IV database; therefore, our study was a *post hoc* analysis of the MIMIC-IV database, the level of evidence was not strong enough, and further high-quality prospective studies are needed to validate the relationship between GLR and sepsis prognosis.

## Conclusion

There was a non-linear relationship between GLR and in-hospital mortality in intensive care patients with sepsis. A higher GLR in ICU patients is associated with in-hospital mortality.

## Data availability statement

The datasets presented in this study can be found in online repositories. The names of the repository/repositories and accession number(s) can be found below: https://mimic.physionet.org/.

## Ethics statement

Ethical review and approval was not required for the study on human participants in accordance with the local legislation and institutional requirements. Written informed consent for participation was not required for this study in accordance with the national legislation and the institutional requirements.

## Author contributions

SC: study design and manuscript writing. CM: modified the manuscript. LeZ and YW: data collection. JC: data interpretation. ZF: statistical analysis. LiZ and CG: project administration. All authors have approved the manuscript and agreed to the submitted version.

## Footnotes

1
http://www.R-project.org

## References

[1] Singer M Deutschman CS Seymour CW Shankar-Hari M Annane D Bauer M The third international consensus definitions for sepsis and septic shock (sepsis-3). *JAMA.* (2016) 315:801–10. 10.1001/jama.2016.0287 26903338 PMC4968574

[2] Hotchkiss RS Moldawer LL Opal SM Reinhart K Turnbull IR Vincent JL. Sepsis and septic shock. *Nat Rev Dis Prim.* (2016) 2:16045. 10.1038/nrdp.2016.45 28117397 PMC5538252

[3] Goh KH Wang L Yeow AYK Poh H Li K Yeow JJL Artificial intelligence in sepsis early prediction and diagnosis using unstructured data in healthcare. *Nat Commun.* (2021) 12:711. 10.1038/s41467-021-20910-4 33514699 PMC7846756

[4] Vincent JL Marshall JC Namendys-Silva SA François B Martin-Loeches I Lipman J Assessment of the worldwide burden of critical illness: the intensive care over nations (ICON) audit. *Lancet Respir Med.* (2014) 2:380–6. 10.1016/S2213-2600(14)70061-X 24740011

[5] Angus DC Barnato AE Bell D Bellomo R Chong CR Coats TJ A systematic review and meta-analysis of early goal-directed therapy for septic shock: the arise, process and promise investigators. *Intensive Care Med.* (2015) 41:1549–60. 10.1007/s00134-015-3822-1 25952825

[6] Rudd KE Johnson SC Agesa KM Shackelford KA Tsoi D Kievlan DR Global, regional, and national sepsis incidence and mortality, 1990-2017: analysis for the Global Burden of Disease Study. *Lancet.* (2020) 395:200–11. 10.1016/S0140-6736(19)32989-7 31954465 PMC6970225

[7] Zhang YY Ning BT. Signaling pathways and intervention therapies in sepsis. *Signal Transduct Target Ther.* (2021) 6:407. 10.1038/s41392-021-00816-9 34824200 PMC8613465

[8] Ankawi G Neri M Zhang J Breglia A Ricci Z Ronco C. Extracorporeal techniques for the treatment of critically ill patients with sepsis beyond conventional blood purification therapy: the promises and the pitfalls. *Crit Care.* (2018) 22:262. 10.1186/s13054-018-2181-z 30360755 PMC6202855

[9] Liu S Li Y She F Zhao X Yao Y. Predictive value of immune cell counts and neutrophil-to-lymphocyte ratio for 28-day mortality in patients with sepsis caused by intra-abdominal infection. *Burns Trauma.* (2021) 9:tkaa040. 10.1093/burnst/tkaa040 33768121 PMC7982795

[10] Moisa E Corneci D Negoita S Filimon CR Serbu A Negutu MI Dynamic changes of the neutrophil-to-lymphocyte ratio, systemic inflammation index, and derived neutrophil-to-lymphocyte ratio independently predict invasive mechanical ventilation need and death in critically Ill COVID-19 patients. *Biomedicines.* (2021) 9:1656. 10.3390/biomedicines9111656 34829883 PMC8615772

[11] Hou SK Lin HA Chen SC Lin CF Lin SF. Monocyte distribution width, neutrophil-to-lymphocyte ratio, and platelet-to-lymphocyte ratio improves early prediction for sepsis at the emergency. *J Pers Med.* (2021) 11:732. 10.3390/jpm11080732 34442376 PMC8402196

[12] Spoto S Lupoi DM Valeriani E Fogolari M Locorriere L Beretta Anguissola G Diagnostic accuracy and prognostic value of neutrophil-to-lymphocyte and platelet-to-lymphocyte ratios in septic patients outside the intensive care unit. *Medicina (Kaunas).* (2021) 57:811. 10.3390/medicina57080811 34441017 PMC8399559

[13] Kriplani A Pandit S Chawla A de la Rosette J Laguna P Jayadeva Reddy S Neutrophil-lymphocyte ratio (NLR), platelet-lymphocyte ratio (PLR) and lymphocyte-monocyte ratio (LMR) in predicting systemic inflammatory response syndrome (SIRS) and sepsis after percutaneous nephrolithotomy (PNL). *Urolithiasis.* (2022) 50:341–8. 10.1007/s00240-022-01319-0 35246692 PMC9110452

[14] Han YQ Zhang L Yan L Li P Ouyang PH Lippi G Red blood cell distribution width predicts long-term outcomes in sepsis patients admitted to the intensive care unit. *Clin Chim Acta.* (2018) 487:112–6. 10.1016/j.cca.2018.09.019 30218659

[15] Fan YW Liu D Chen JM Li WJ Gao CJ. Fluctuation in red cell distribution width predicts disseminated intravascular coagulation morbidity and mortality in sepsis: a retrospective single-center study. *Minerva Anestesiol.* (2021) 87:52–64. 10.23736/S0375-9393.20.14420-1 33538418

[16] Dankl D Rezar R Mamandipoor B Zhou Z Wernly S Wernly B Red cell distribution width is independently associated with mortality in sepsis. *Med Princ Pract.* (2022) 31:187–94. 10.1159/000522261 35093953 PMC9209973

[17] Cao C Yu M Chai Y. Pathological alteration and therapeutic implications of sepsis-induced immune cell apoptosis. *Cell Death Dis.* (2019) 10:782. 10.1038/s41419-019-2015-1 31611560 PMC6791888

[18] Nakamori Y Park EJ Shimaoka M. Immune deregulation in sepsis and septic shock: reversing immune paralysis by targeting PD-1/PD-L1 pathway. *Front Immunol.* (2020) 11:624279. 10.3389/fimmu.2020.624279 33679715 PMC7925640

[19] Brakenridge SC Moore FA Mercier NR Cox M Wu Q Moldawer LL Persistently elevated glucagon-like peptide-1 levels among critically Ill surgical patients after sepsis and development of chronic critical illness and dismal long-term outcomes. *J Am Coll Surg.* (2019) 229:58–67.e1. 10.1016/j.jamcollsurg.2019.04.014 30991107 PMC6599553

[20] Jiang L Cheng M. Impact of diabetes mellitus on outcomes of patients with sepsis: an updated systematic review and meta-analysis. *Diabetol Metab Syndr.* (2022) 14:39. 10.1186/s13098-022-00803-2 35248158 PMC8898404

[21] Li X Liu C Mao Z Xiao M Wang L Qi S Predictive values of neutrophil-to-lymphocyte ratio on disease severity and mortality in COVID-19 patients: a systematic review and meta-analysis. *Crit Care.* (2020) 24:647. 10.1186/s13054-020-03374-8 33198786 PMC7667659

[22] Huang Z Fu Z Huang W Huang K. Prognostic value of neutrophil-to-lymphocyte ratio in sepsis: a meta-analysis. *Am J Emerg Med.* (2020) 38:641–7. 10.1016/j.ajem.2019.10.023 31785981

[23] Mitsuyama Y Shimizu K Komukai S Hirayama A Takegawa R Ebihara T Sepsis-associated hypoglycemia on admission is associated with increased mortality in intensive care unit patients. *Acute Med Surg.* (2022) 9:e718. 10.1002/ams2.718 35106180 PMC8785236

[24] Li L Zou G Liu J. Preoperative glucose-to-lymphocyte ratio is an independent predictor for acute kidney injury after cardiac surgery in patients in intensive care unit. *Int J Gen Med.* (2021) 14:6529–37. 10.2147/IJGM.S335896 34675620 PMC8518472

[25] Navarro J Kang I Hwang HK Yoon DS Lee WJ Kang CM. Glucose to lymphocyte ratio as a prognostic marker in patients with resected pT2 gallbladder cancer. *J Surg Res.* (2019) 240:17–29. 10.1016/j.jss.2019.02.043 30909062

[26] Zhong A Cheng CS Kai J Lu R Guo L. Clinical significance of glucose to lymphocyte ratio (GLR) as a prognostic marker for patients with pancreatic cancer. *Front Oncol.* (2020) 10:520330. 10.3389/fonc.2020.520330 33117673 PMC7561421

[27] Chen Y Tang S Wang Y. Prognostic value of glucose-to-lymphocyte ratio in critically Ill patients with acute pancreatitis. *Int J Gen Med.* (2021) 14:5449–60. 10.2147/IJGM.S327123 34526812 PMC8436258

[28] Liu T Zhao Q Du B. Effects of high-flow oxygen therapy on patients with hypoxemia after extubation and predictors of reintubation: a retrospective study based on the MIMIC-IV database. *BMC Pulm Med.* (2021) 21:160. 10.1186/s12890-021-01526-2 33985472 PMC8118109

[29] von Elm E Altman DG Egger M Pocock SJ Gøtzsche PC Vandenbroucke JP. Strengthening the reporting of observational studies in epidemiology (strobe) statement: guidelines for reporting observational studies. *BMJ.* (2007) 335:806–8. 10.1136/bmj.39335.541782.AD 17947786 PMC2034723

[30] Komorowski M Celi LA Badawi O Gordon AC Faisal AA. The artificial intelligence clinician learns optimal treatment strategies for sepsis in intensive care. *Nat Med.* (2018) 24:1716–20. 10.1038/s41591-018-0213-5 30349085

[31] Yang Q Zheng J Chen W Chen X Wen D Chen W Association between preadmission metformin use and outcomes in intensive care unit patients with sepsis and type 2 diabetes: a cohort study. *Front Med (Lausanne).* (2021) 8:640785. 10.3389/fmed.2021.640785 33855034 PMC8039324

[32] Feng M McSparron JI Kien DT Stone DJ Roberts DH Schwartzstein RM Transthoracic echocardiography and mortality in sepsis: analysis of the MIMIC-III database. *Intensive Care Med.* (2018) 44:884–92. 10.1007/s00134-018-5208-7 29806057

[33] Preau S Vodovar D Jung B Lancel S Zafrani L Flatres A Energetic dysfunction in sepsis: a narrative review. *Ann Intensive Care.* (2021) 11:104. 10.1186/s13613-021-00893-7 34216304 PMC8254847

[34] Chang MW Huang CY Liu HT Chen YC Hsieh CH. Stress-induced and diabetic hyperglycemia associated with higher mortality among intensive care unit trauma patients: cross-sectional analysis of the propensity score-matched population. *Int J Environ Res Public Health.* (2018) 15:992. 10.3390/ijerph15050992 29762485 PMC5982031

[35] Ali Abdelhamid Y Kar P Finnis ME Phillips LK Plummer MP Shaw JE Stress hyperglycaemia in critically ill patients and the subsequent risk of diabetes: a systematic review and meta-analysis. *Crit Care.* (2016) 20:301. 10.1186/s13054-016-1471-6 27677709 PMC5039881

[36] Cui Y Chen W Chi J Wang L. Comparison of transcriptome between type 2 diabetes mellitus and impaired fasting glucose. *Med Sci Monit.* (2016) 22:4699–706. 10.12659/MSM.896772 27906902 PMC5147684

[37] Wang Z Ren J Wang G Liu Q Guo K Li J. Association between diabetes mellitus and outcomes of patients with sepsis: a meta-analysis. *Med Sci Monit.* (2017) 23:3546–55. 10.12659/MSM.903144 28727676 PMC5533197

[38] Marik PE Bellomo R. Stress hyperglycemia: an essential survival response! *Crit Care.* (2013) 17:305. 10.1186/cc12514 23470218 PMC3672537

[39] Monserrat J de Pablo R Reyes E Díaz D Barcenilla H Zapata MR Clinical relevance of the severe abnormalities of the T cell compartment in septic shock patients. *Crit Care.* (2009) 13:R26. 10.1186/cc7731 19243622 PMC2688144

[40] Monserrat J de Pablo R Diaz-Martín D Rodríguez-Zapata M de la Hera A Prieto A Early alterations of B cells in patients with septic shock. *Crit Care.* (2013) 17:R105. 10.1186/cc12750 23721745 PMC4056890

[41] Drewry AM Samra N Skrupky LP Fuller BM Compton SM Hotchkiss RS. Persistent lymphopenia after diagnosis of sepsis predicts mortality. *Shock.* (2014) 42:383–91. 10.1097/SHK.0000000000000234 25051284 PMC4362626

[42] Heffernan DS Monaghan SF Thakkar RK Machan JT Cioffi WG Ayala A. Failure to normalize lymphopenia following trauma is associated with increased mortality, independent of the leukocytosis pattern. *Crit Care.* (2012) 16:R12. 10.1186/cc11157 22264310 PMC3396248

[43] Girardot T Rimmelé T Venet F Monneret G. Apoptosis-induced lymphopenia in sepsis and other severe injuries. *Apoptosis.* (2017) 22:295–305. 10.1007/s10495-016-1325-3 27812767

[44] Liu Y Zheng J Zhang D Jing L. Neutrophil-lymphocyte ratio and plasma lactate predict 28-day mortality in patients with sepsis. *J Clin Lab Anal.* (2019) 33:e22942. 10.1002/jcla.22942 31265174 PMC6757133

[45] Shen Y Huang X Zhang W. Platelet-to-lymphocyte ratio as a prognostic predictor of mortality for sepsis: interaction effect with disease severity-a retrospective study. *BMJ Open.* (2019) 9:e022896. 10.1136/bmjopen-2018-022896 30782690 PMC6352809

[46] Zhang Y Xu Y Wang D Kuang T Wu W Xu X Prognostic value of preoperative glucose to lymphocyte ratio in patients with resected pancreatic cancer. *Int J Clin Oncol.* (2021) 26:135–44. 10.1007/s10147-020-01782-y 32959232

