# Supplementary material for: Association between glucose-to-lymphocyte ratio and in-hospital mortality in intensive care patients with sepsis: A retrospective observational study based on Medical Information Mart for Intensive Care IV
Source: Front Med (Lausanne). 2022 Aug 24;9:922280. doi: 10.3389/fmed.2022.922280 (PMC9448903; doi:10.3389/fmed.2022.922280)
Supplement: Supplementary file 1 [file Table_1.pdf]

TABLE S1 | Sensitivity analysis of patients after excluding missing data from the study.

Variable	n	Unadjusted		Model 1		Model 2		Model 3	
		HR_95CI%	*P*-value	HR_95CI%	*P*-value	HR_95CI%	*P*-value	HR_95CI%	*P*-value
GLR	7756	1.11 (1.09~1.13)	<0.001	1.125 (1.09~1.13)	<0.001	1.06 (1.04~1.08)	<0.001	1.03 (1.0~1.05)	0.020
GLR4									
Q1(GLR<0.43)	1867	1(Ref)		1(Ref)		1(Ref)		1(Ref)	
Q2(0.43≤GLR<0.78)	1964	1.23 (1.01~1.48)	0.036	1.23 (1.019~1.487)	0.031	1.24 (1.03~1.5)	0.027	1.19 (0.98~1.44)	0.074
Q3(0.78≤GLR<1.56)	1911	1.72 (1.45~2.05)	<0.001	1.70 (1.43~2.02)	<0.001	1.49 (1.25~1.78)	<0.001	1.306 (1.094~1.56)	0.003
Q4(GLR ≥1.56)	2014	2.41 (2.04~2.85)	<0.001	2.34 (1.98~2.76)	<0.001	1.74 (1.47~2.07)	<0.001	1.396 (1.17~1.66)	<0.001
P for trend.test			<0.001		<0.001		<0.001		<0.001

Model 1 = Adjust for (Age+ sex)

Model 2 = Model 1+ (ethnicity +weight + MAP + HR +SPO2+ hemoglobin + PLT + WBC + lactate + pH).

Model 3 = Model 2+ (SOFA score+ APS III + ventilator use+ diabetes +CCI+ vasopressin use).
